# An Illustrated Dictionary of Medicine, Biology, and Allied Sciences

**Published:** 1894-08

**Authors:** 


### An Illustrated Dictionary of Medicine, Biology, and Allied Sciences. By George M. Gould, A. M., M. D.

This book was reviewed in the July number of this journal, but the prices were not given. The publishers have furnished this desirable item of information and consequently we are able to state them for the benefit of our readers.

They are as follow: Half morocco, $10; full sheep, $10; half Russia, indexed, $12. All these prices are net.

